# Preventive effect of a heparinoid-containing product on the application site reaction of the rotigotine transdermal patch in Parkinson’s disease: A pilot randomized clinical trial (the SkinHeRo study)

**DOI:** 10.1016/j.prdoa.2021.100105

**Published:** 2021-08-13

**Authors:** Jinsoo Koh, Maiko Takahashi, Mayumi Sakata, Masaaki Yasui, Shoko Yorozu, Hidefumi Ito

**Affiliations:** Department of Neurology, Wakayama Medical University, Japan

**Keywords:** AE, adverse event, Parkinson’s disease, Skin, Moisturizing, Quality of life, dopamine agonist, Patch

## Abstract

**Introduction:**

The rotigotine transdermal patch (RTP) is a dopamine agonist used to treat Parkinson’s disease (PD) but is sometimes discontinued because of application site reactions (ASRs). We aimed to investigate the effect of a heparinoid-containing product (HCP) for preventing ASRs due to the RTP by conducting a randomized controlled pilot trial.

**Methods:**

Twenty patients with idiopathic non-demented PD were randomized to the skin care group using a HCP (group H) and the non-skin care group (group N). The primary outcome was the change in the baseline Skindex-16 score (ΔSkindex-16) at week 4. In addition, skin symptoms were also evaluated using the Dermatology Life Quality Index (DLQI) and International Contact Dermatitis Research Group (ICDRG) system for clinical scoring allergic patch test reactions up to week 8.

**Results:**

The ΔSkindex-16 score at week 4 tended to be lower in group H than in group N, although the difference was not statistically significant (−1.5 ± 2.0 vs 1.3 ± 10.9, p = 0.53). When the patients with baseline Skindex-16 scores ≥ 7 were excluded, the ΔSkindex-16 at week 4 was significantly lower in group H (−1.5 ± 2.0 vs 6.1 ± 8.6, p = 0.042). The DLQI also tended to be lower in group H at weeks 4 and 8, but not significantly (p = 0.066 and p = 0.077, respectively). The ICDRG score at week 4 was significantly lower in group H (p = 0.044).

**Conclusion:**

We suggest that the HCP has a preventive effect against ASRs cause by the RTP.

## Highlights

• This is the first report on the inhibitory effect of HCP on ASRs caused by the RTP.
• We provided Skindex-16 scores for PD with the RTP.
• Appropriate skincare using HCP can prevent the ASRs induced by the RTP.

## 1 Introduction

Parkinson’s disease (PD) is the second most common neurodegenerative disease. As still no radical treatment has been established, the main patient care is symptomatic treatment with dopamine replacement therapy. Although levodopa has played a central role in dopamine replacement therapy, dopamine agonists are also needed as treatment options owing to their benefits from their levodopa-sparing effects, continuous dopaminergic stimulation, and ability to improve depression or apathy [1], [2]. Among dopamine agonists, the rotigotine transdermal patch (RTP) can particularly achieve continuous dopaminergic stimulation by continuous transdermal absorption. Furthermore, because its dopamine receptor stimulation profile is similar to that of dopamine [3], it is expected to have fewer side effects than other dopamine agonists.

Although rotigotine has various uses, its clinical use has a specific problem of application site reactions (ASRs). A previous study revealed that 44% of patients experienced ASRs [4]. Neurologists often encounter ASRs that cause discontinuation of RTP use. In general, ASRs by transdermal preparation can be divided into “physical” and “chemical” irritations. ASRs can sometimes occur as allergic reactions to rotigotine but are often caused by exfoliation of the keratin or mechanical irritation. Especially in elderly patient with PD, the skin barrier mechanism is reduced, which leads to an increase in the occurrences of skin disorders. Therefore, appropriate skin care is important to prevent and reduce ASRs. However, this has not been thoroughly investigated.

Heparinoid-containing products (HCPs) have long been used to manage various skin conditions. HCPs increased stratum corneum hydration and accelerated permeability barrier recovery in mice and young and aged humans [5]. Topical glucocorticoids are also used for ASRs caused by rotigotine [6] but can induce atrophogenic side effects during long-term use. Recently, a topical HCP has also been reported to have a preventive effect against glucocorticoid-induced alterations of the epidermal permeability barrier homeostasis and atrophy [7].

Although HCPs might be useful agents for reducing ASRs caused by the RTP, only few clinical studies have been conducted on the topic so far [8]. Thus, in present study, we aimed to investigate the effect of a HCP for preventing ASRs due to RTP by conducting a randomized control pilot trial.

## 2 Methods

### 2.1 Study design

This was a pilot study conducted to examine the efficacy and safety of HCPs for the prevention of ASRs by the RTP in patients with PD. Patients who were indicated to receive rotigotine in clinical use were randomly assigned to the skin care group using a HCP (group H), and those who did not receive heparinoids were assigned to the non-skin care group (group N), and the severity of skin symptoms was compared. The rotigotine dose was adjusted for normal clinical use. In an 8-week clinical trial, clinical symptoms were evaluated at weeks 1, 4, and 8 after the start of the rotigotine therapy. This study was conducted in accordance with the principles of the Declaration of Helsinki and approved by the Wakayama Medical University Ethics Committee (approval No. 2373) and was registered at the University hospital Medical Information Network (UMIN) clinical trials registry (UMIN000033409).

### 2.2 Participants

We consecutively enrolled 20 patients with idiopathic PD. The exclusion criteria were (1) severe dementia that caused inability to answer the questionnaire accurately, (2) any severe skin disorders that affect the determination of the ASRs by rotigotine, (3) past history of severe allergic reactions to rotigotine or HCP, and (4) patients who were judged as unsuitable for participation in this study. All the participants provided written informed consent.

### 2.3 Intervention

In group H, HCP (Hirudoid cream 0.3%, Maruho, Osaka, Japan) was applied once daily on the sites (shoulder, upper arm, abdomen, thigh, and buttocks on both sides) where RTPs were to be applied. HCP application was started 1 week before rotigotine use. The patients were instructed to use the HCP in sufficient amounts as follows: one fingertip unit (approximately 0.5 g) for the area on both palms, or approximately 25 g/week. In group N, no HCP was used. The participants were prohibited to use any other skin care products on the sites where RTPs were to be applied and other new patches. For randomization, we used the permuted block method without adjustment factors and the randomization list was generated from code in statistical software R (https://www.R-project.org/).

### 2.4 Outcome measures

The primary outcome was the change in the baseline Skindex-16 [9] score (ΔSkindex-16) at week 4. Skindex-16 is a brief version of Skindex, which is a questionnaire that measures the effects of skin diseases on patient quality of life (QOL), consisting of 16 questions presented as a seven-grade scale. The responses were then transformed to a 100-point linear scale ranging from 0 (never bothered) to 100 (always bothered), and a global score was calculated by averaging the scores of each item. As a secondary outcome, the Skindex-16 and ΔSkindex-16 scores at each visit were also evaluated.

Dermatology Life Quality Index (DLQI) score [10] is also an evaluation index of QOL specialized for skin diseases. The total score (0–30 points) of each item is calculated, and the higher the score, the worse the QOL. The International Contact Dermatitis Research Group (ICDRG) system for clinical scoring of allergic patch test reactions [11] was also used to assess for objective macroscopic findings. The ICDRG score was evaluated on a 6-point scale according to the skin condition of the most recent rotigotine application site as follows: negative reaction (−), doubtful reaction (?+), weak (non‐vesicular) positive reaction (+), strong (vesicular) positive reaction (++), extreme positive reaction (+++), and irritant reaction (IR). For evaluation of the PD symptoms, the unified PD Rating Scale (UPDRS) and PD Questionnaire-39 (PDQ-39) were used.

### 2.5 Statistical analysis

Statistical analysis was performed using the JMP Pro 14 software. Continuous variables are presented as mean ± standard deviation and 95% confidence intervals. Categorical variables are presented as numerals. For group comparisons, the Student *t* test or Fisher exact test was performed, depending on the type of variable.

## 3 Results

### 3.1 Participants

All 20 potential participants screened met the eligibility criteria. Both groups were randomly allocated with 10 participants. Both groups were similar in demographic and clinical characteristics at baseline, although the mean age and proportion of women tended to be higher in group H (Table 1). The participants randomized to group H used sufficient heparinoid doses as directed throughout the trial (2.4 ± 1.0 g/day). Four participants dropped out of the trial for the following reasons: withdrawal of consent before the start of RTP application, admission to another hospital because of pelvic fracture, exacerbation of preexisting depression and irritation even without ASRs, and exacerbation of pitting edema due to the adverse effect of rotigotine (Supplementary Fig. 1). No participants used any topical corticosteroids or other skin care products during the study period. No other adverse effects were reported.

**Table 1:** Demographic characteristics of the patients.

	Heparinoid group	Non-skin care group	p
Age, years	72.5 ± 8.2	70.6 ± 7.7	0.60
Sex, female/male	6/4	2/8	0.17
UPDRS part 3	20.3 ± 8.7	24.3 ± 9.3	0.34
Skindex-16	2.4 ± 2.8	4.8 ± 6.7	0.30
DLQI	0.6 ± 0.7	1.2 ± 1.5	0.27
PDQ-39 summary index	25.0 ± 17.6	25.2 ± 13.5	0.98

### 3.2 Skindex-16

The primary outcome, the ΔSkindex-16 score, and the Skindex-16 score at week 4 tended to be lower in group H than in group N, although not statistically significantly (Table 2). When the patients with baseline Skindex-16 scores ≥ 7 were excluded to eliminate the effects of other skin symptoms, the ΔSkindex-16 score was significantly lower in group H as follows: H (n = 7), −1.5 ± 2.0 [−3.3, 0.3]; N (n = 7), 6.1 ± 8.6 [−1.9, 14.1]; p = 0.042, t = 2.3. At week 8, both the ΔSkindex-16 and Skindex-16 scores also tended to be lower in group H (Table 2). Even at week 1, excluding one case of outlier (the case of dropout before week 4 because of depression: Skindex-16 score of 53 at week 1 without ASRs), the Skindex-16 score also tended to be lower in H group (1.2 ± 2.2 vs 7.4 ± 10.4, p = 0.11).

**Table 2:** Primary and secondary outcomes.

	Heparinoid group	Non-skin care group	p
ΔSkindex-16			
Week 1	4.5 ± 19.3 [−10.3, 19.3]	2.6 ± 9.0 [−3.9, 9.1]	0.78
Week 4	−1.5 ± 2.0 [−3.3, 0.3]	1.3 ± 10.9 [−6.6, 9.1]	0.53
Week 8	0.0 ± 4.8 [−4.4, 4.4]	1.7 ± 7.8 [−4.3, 7.7]	0.62
Skindex-16			
Week 1	7.2 ± 18.1 [−6.7, 21.1]	7.4 ± 10.4 [−0.0, 14.8]	0.97
Week 4	0.7 ± 1.0 [−0.2, 1.7]	6.0 ± 6.7 [1.1, 10.9]	0.063
Week 8	2.2 ± 5.9 [−3.2, 7.7]	6.6 ± 5.7 [2.2, 11.0]	0.15
DLQI			
Week 1	1.3 ± 2.6 [−0.7, 3.3]	1.4 ± 1.4 [0.4, 2.4]	0.94
Week 4	0.4 ± 0.5 [−0.1, 0.9]	1.2 ± 0.9 [0.5, 1.9]	0.066
Week 8	0.3 ± 0.8 [−0.4, 1.0]	1.1 ± 0.9 [0.4, 1.8]	0.077
ICDRG, -/?+			
Week 1	6/3	8/2	0.63
Week 4	7/0	5/5	0.044*
Week 8	6/1	8/1	1.00
UPDRS motor score			
Week 4	14.1 ± 6.6 [8.0, 20.3]	20.1 ± 9.5 [13.3, 26.9]	0.18
Week 8	10.7 ± 6.5 [4.7, 16.7]	20.2 ± 9.7 [12.8, 27.7]	0.043*
PDQ-39 summary index			
Week 4	11.5 ± 7.1 [4.9, 18.0]	22.8 ± 13.5 [13.2, 32.5]	0.060
Week 8	11.3 ± 9.5 [2.4, 20.1]	18.1 ± 14.3 [7.1, 29.1]	0.29
Rotigotine, mg/24 h			
Week 1	2.2 ± 0.6 [1.7, 2.7]	2.0 ± 0.0 [2.0, 2.0]	0.31
Week 4	2.9 ± 1.1 [1.9, 3.8]	2.8 ± 1.0 [2.1, 3.5]	0.88
Week 8	3.7 ± 1.4 [2.4, 5.0]	3.8 ± 1.6 [2.6, 5.0]	0.93

*p < 0.05.

### 3.3 Other secondary outcomes

The DLQI also tended to be lower in group H at weeks 4 and 8, although the difference was not statistically significant (Table 2). The ICDRG score did not show any positive or higher values over the whole period. The number of cases (percent) in which a ICDRG score of doubtful reaction (?+) was observed at least once during the entire period after the start of rotigotine administration was 4 (44%) of the 9 patients in group H and 6 (86%) of the 7 patients in group N. The rate of doubtful reaction was significantly higher in group H at week 4 (Table 2).

The rotigotine doses were increased after week 8 and were not different between the two groups in any of the visits. The rotigotine dose was relatively small, approximately 4 mg/24 h on average even in week 8, and the maximum dose was 6 mg/24 h. After the start of rotigotine application, the UPDRS motor and PDQ-39 scores decreased in both groups. The UPDRS motor score at week 8 was significantly lower in group H than in group N.

## 4 Discussion

This is the first study to investigate the preventive effect of HCP on ASRs caused by the RTP. The primary end point, ΔSkindex-16 score at week 4, was significantly lower in group H when inappropriate cases were excluded. Other indicators such as DLQI and ICDRG score also showed a tendency for less ASRs in group H. As no previous studies have investigated the preventive effect of HCPs for ASRs, the present study did not estimate the appropriate sample size and was conducted as a pilot study. As a result, the sample size was small, and some clinical indicators showed a tendency but could not show a statistically significant difference. Another reason is that the rotigotine dose was less than expected, so only few ASRs and the flooring effect occurred. That is, even a low dose of rotigotine could unexpectedly show a sufficient effect for improving clinical symptoms.

Age and sex differed, though not significantly, between the groups. In this study, the HCP group tended to be older. As the skin barrier function is generally suggested to decrease with aging, the fact that fewer skin disorders occurred in group H is not considered to be an effect of age. Sex-related differences in the adverse effects of the medication are still controversial [12], but at least randomized controlled trials of rotigotine have not pointed out sex-related differences in ASRs [4].

The cause of the significantly higher effect of improving motor symptoms in group H is unknown but highly probable to be a coincidence. However, the potential of HCPs to improve rotigotine absorption by reducing skin disorders, resulting in improved motor symptoms, is undeniable but impossible to prove in this study because we did not measure the blood concentration of rotigotine.

This study has some other limitations. The present study was open-label, and placebo effect could not be ruled out. As mentioned earlier, the maximum rotigotine dose is as low as 6 mg/24 h, and the effect of HCPs at high doses of rotigotine is unknown. Finally, it was an 8-week short-term study, so long-term effects could not be determined.

Transdermal preparations have merits such as stable blood concentration and independence on swallowing function. Therefore, transdermal preparations may be further developed for the treatment of various diseases. In this context, controlling ASRs is important for improving medication adherence, particularly in elderly patients. The HCP skin care method in this study may be applicable when using other patches, and future studies are needed.

In conclusion, we suggest that the HCP introduced in this study have preventive effects against ASRs caused by the RTP. For verification, a placebo-controlled randomized controlled trial is required after estimating the sample size based on this study.

## CRediT authorship contribution statement

**Jinsoo Koh:** Conceptualization, Methodology, Investigation, Validation, Writing – original draft, Project administration, Funding acquisition. **Maiko Takahashi:** Methodology, Validation, Data curation. **Mayumi Sakata:** Investigation. **Masaaki Yasui:** Investigation. **Shoko Yorozu:** Methodology. **Hidefumi Ito:** Writing - review & editing, Supervision.

## Declaration of Competing Interest

The authors declare that they have no known competing financial interests or personal relationships that could have appeared to influence the work reported in this paper.
