# Supplementary material for: Preventive effect of a heparinoid-containing product on the application site reaction of the rotigotine transdermal patch in Parkinson’s disease: A pilot randomized clinical trial (the SkinHeRo study)
Source: Clin Park Relat Disord. 2021 Aug 13;5:100105. doi: 10.1016/j.prdoa.2021.100105 (PMC8379504; doi:10.1016/j.prdoa.2021.100105)
Supplement: Supplementary data 1 [file mmc1.pdf]

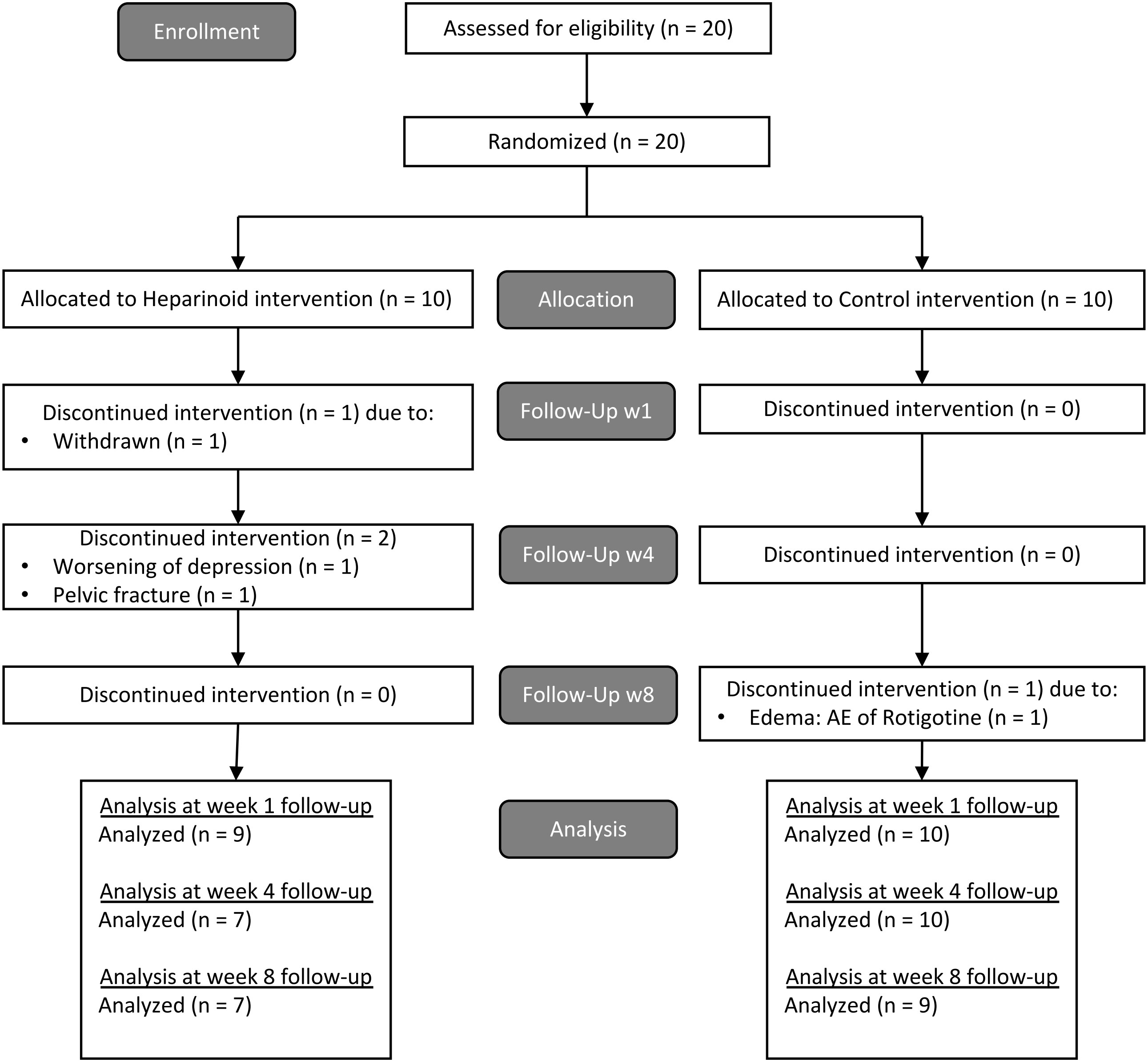
Enrollment
Assessed for eligibility (n = 20)
Randomized (n = 20)
Allocated to Heparinoid intervention (n = 10)
Allocation
Allocated to Control intervention (n = 10)
Discontinued intervention (n = 1) due to:
• Withdrawn (n = 1)
Follow-Up w1
Discontinued intervention (n = 0)
Discontinued intervention (n = 2)
• Worsening of depression (n = 1)
• Pelvic fracture (n = 1)
Follow-Up w4
Discontinued intervention (n = 0)
Discontinued intervention (n = 0)
Follow-Up w8
Discontinued intervention (n = 1) due to:
• Edema: AE of Rotigotine (n = 1)
Analysis at week 1 follow-up
Analyzed (n = 9)
Analysis at week 4 follow-up
Analyzed (n = 7)
Analysis at week 8 follow-up
Analyzed (n = 7)
Analysis
Analysis at week 1 follow-up
Analyzed (n = 10)
Analysis at week 4 follow-up
Analyzed (n = 10)
Analysis at week 8 follow-up
Analyzed (n = 9)
